# How to put plant root uptake into a soil water flow model: A documentation with complete computer code

**DOI:** 10.12688/f1000research.7686.1

**Published:** 2016-01-08

**Authors:** Xuejun Dong

**Affiliations:** 1 North Dakota State University, Central Grassland Research Extension Center, Streeter, USA; 2 Texas A&M AgriLife Research, Uvalde, USA

**Keywords:** Computer simulation, Richards’ equation, Root growth, Soil water balance, Transpiration, Uptake compensation, Water stress

## Abstract

The need for improved crop water use efficiency calls for flexible modeling platforms to implement new ideas in plant root uptake and its regulation mechanisms. This paper documents the details of modifying a soil infiltration and redistribution model to include (a) dynamic root growth, (b) non-uniform root distribution and water uptake, (c) the effect of water stress on plant water uptake, and (d) soil evaporation. The paper also demonstrates strategies of using the modified model to simulate soil water dynamics and plant transpiration considering different sensitivity of plants to soil dryness and different mechanisms of root water uptake. In particular, the flexibility of simulating various degrees of compensated uptake (whereby plants tend to maintain potential transpiration under mild water stress) is emphasized. The paper also describes how to estimate unknown root distribution and rooting depth parameters by the use of a simulation-based searching method. The full documentation of the computer code will allow further applications and new development.

## Introduction

Increased biomass and yield of agricultural crops hinges on improved efficiency of root water uptake ^1, 2^. Biophysical mechanisms responsible for the improved uptake efficiency in turn rely on linkages between plant physiological processes and the physics of soil water movement; the development of the latter has been well illustrated in 3, 4 and 5. Research literature in this and closely related areas has been profound in past decades, though roughly two categories of approach can be identified: one that is mechanistic and one that is phenomenological in nature. The mechanistic approach has improved our understanding of the soil-root interactions by identifying major resistances and driving forces of water flow from soil to roots ^3, 4^, as well as the scale-dependent interactions and geometry among soil organisms responsible for water and nutrient uptake ^6, 7^. Yet effective mechanistic modeling needs a large amount of empirical data support, which can not always be met due to various factors constraining research activities. On the other hand, an intuitive, phenomenological approach can be implemented with less empirical data support. Examples of this latter approach may include constraining root water uptake capacity by soil water potential ^8^, by root length distribution ^9– 11^, and through additional modifications of the root uptake sensitivity to local soil water potential and root density ^12– 16^, which allows compensated uptake to maintain potential transpiration ^17, 18^.

One of the major goals of agronomic management for increased water use efficiency is to channel water loss through transpiration ^19^. This to some extent can be aided by the compensated root uptake, which has been implemented in software such as HYDRUS ( http://www.pc-progress.com/en/Default.aspx?hydrus-1d) through the use of a threshold root uptake adaptation factor, whereby reduced water uptake in water-stressed parts of the root zone is fully compensated for by increased uptake in other soil regions where water is available ^20^.

However, root water uptake compensation has been implemented in different studies using different models, which naturally calls for the flexibility of implementation of various forms of the compensation mechanism in root uptake studies. The first objective of this paper is to show that this flexibility can be obtained by using a water flow model extended from an original model as described in 5. This extended model was used in generating numerical solutions of water flow problems in 16.

The second objective of this paper is to provide a tool for quantifying plant water uptake under conditions where information of root depth and distribution pattern is not known. This is relevant to agricultural applications as fine root information remains difficult to obtain despite the progress made in advanced underground sensing ^21^. We take advantage of inverse modeling, similar to 22 and 10, to find optimal solutions of unknown root parameters based on good measurement of soil water content, which can be obtained through the use of various soil moisture sensors. Despite excellent documentation of the methods for solving the soil water flow equations relevant to agricultural water management ^3, 5^, the author has not seen a paper documenting the details of how to put the root uptake component into a soil water flow model, as well as providing the details of a search-based optimization procedure for root parameter estimation.

## Methods

### Implementation

The one dimensional Richards equation of soil water flow in the vertical direction can be written as

$$c\frac{\partial h}{\partial t}=\frac{\partial }{\partial z}[K\frac{\partial h}{\partial z}]-\frac{\partial K}{\partial z},\;(1)$$

where *h* is water pressure head (m), *t* is time (s), *z* space (m, positive downward and soil surface as zero), *K* hydraulic conductivity (m/s) and $c=\frac{\partial \theta }{\partial h}.$ The reason why there is a negative sign in Equation 1 is that, when we take the space derivative for the gravity potential head *z*, namely, in ∂ *z*/∂ *z*, the values in the numerator change in the opposite direction as that in the denominator: gravity head always decreases downwards but our chosen space *z* increases downwards.

The model described by Equation 1 does not consider a sink of plant root water uptake. It can be solved using the implicit finite difference method as described by 5. Here is a summary of key points with a few intuitive comments. Let’s for now assume surface fluxes as precipitation, irrigation, or surface evaporation. As Equation 1 considers soil water dynamics both in space (vertical axis) and time, we need to properly discretize the equation considering both space and time. Here we let all *h*’s that take space derivatives be approximated using values at the old ( *t* = *n*) and the new ( *t* = *t*_*n*+1_) times, but let *K*’s be approximated in old time ( *t* = *n*). All other variables not taking partial derivatives are to be approximated in the old time. Assume the vertical axis is equally divided by Δ *z*, to approximate Equation 1 at *z* = *z*_*i*_ and *t*_*n*+1_ = *t*_*n*_+Δ *t*, we have the following formula to use:

$$\frac{c_{i,n}(h_{i}^{*}-h_{i,n})}{\Delta t}=\frac{K_{i+0.5,n}(h_{i+1}^{*}-h_{i}^{*})}{{\left(\Delta z\right)}^{2}}-\frac{K_{i-0.5,n}(h_{i}^{*}-h_{i-1}^{*})}{{\left(\Delta z\right)}^{2}}-\frac{\left(K_{i+0.5,n}-K_{i-0.5,n}\right)}{\Delta z}.\;(2)$$

**Figure 1.**
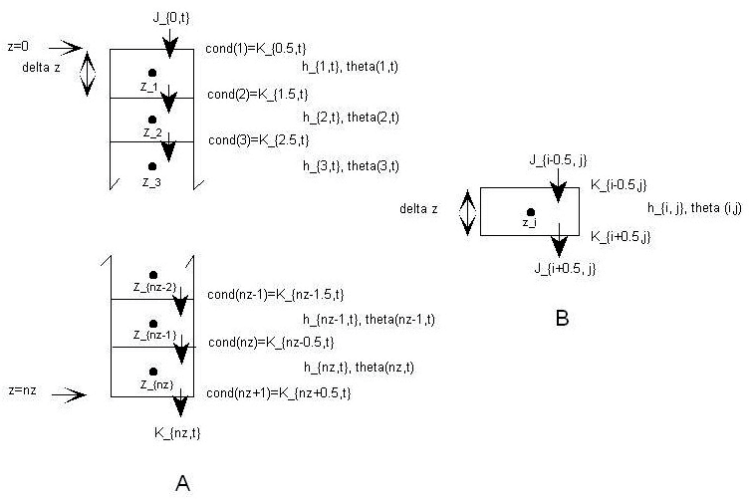
Block-centered finite difference scheme for numerical solution of 1-D Richards equation. **A**. Pressure heads and water conductivities are indexed differently. **B**. Highlight of one internal block. Modified based on Figure 5–13 of 5.

Another interesting aspect of Equation 2 is that the space index for *K* is shifted to the upper edge of each of the blocks, while the index of *h* or *θ* is located at the center of the blocks. This is the characteristic of the *block-centered* finite difference scheme and it will enable the calculation of fluxes between two adjacent soil layers, or blocks, as seen in the right sub-figure of Figure 1, which is based on Figure 5–13 of Warrick’s book (on page 195). In Figure 1, we reinforce the idea that, similar to the situation of conductivity, *K*, the index for flux density also is located at the edge of the blocks. Intuitively, this makes sense, because in the head-based equation of soil water flow, *K* and *J* have the same unit (both are in m/s). As a result, we can compare the magnitude of *K* and *J* directly. For example, if the water conductivity of the surface soil is similar to the rate of precipitation, we can roughly say that all the rain water would have time to fully infiltrate into the soil before ponding on the soil surface.

The terms of the implicit scheme can be collected to form a *tridiagonal* system of linear equations, with the water potentials for the new time (starred) arranged at the left side and those for the old time on the right side of the equation. Then, terms for different space segments can be collected to form the following equation:

$$A_{i}h_{i-1}^{*}+B_{i}h_{i}^{*}+C_{i}h_{i+1}^{*}=D_{i},\;i=1,\ldots ,nz,\;(3)$$

which can be written in matrix form as

$$\left[\begin{array}{lllllll} B_{1} & C_{1} & 0 & \cdots & 0 & 0 & 0\\ A_{2} & B_{2} & C_{2} & \cdots & 0 & 0 & 0\\ \vdots & \vdots & \vdots & \ddots & \vdots & \vdots & \vdots \\ 0 & 0 & 0 & \cdots & A_{nz-1} & B_{nz-1} & C_{nz-1}\\ 0 & 0 & 0 & \cdots & 0 & A_{nz} & B_{nz} \end{array}\right]\times \left[\begin{array}{l} h_{1}^{*}\\ h_{2}^{*}\\ \vdots \\ h_{nz-1}^{*}\\ h_{nz}^{*} \end{array}\right]=\left[\begin{array}{l} D_{1}\\ D_{2}\\ \vdots \\ D_{nz-1}\\ D_{nz\cdot } \end{array}\right]\;(4)$$

Equation 4 is called tridiagonal because, for the coefficient of the *nz* by *nz* square matrix, the elements are all zero except for those located along the three diagonals. From Equation 4, we can see that *A*_1_ = *C*_*nz*_ = 0. Actually, values of the coefficients on the left side of Equation 4 should be determined considering the boundary conditions at the soil surface and at the bottom of the root zone.

Detailed solution procedures of the above model “Austere-Layered" (hereafter referred to as AL.f) are provided in 5. Here in order to let this soil water flow model simulate root uptake, the following new components have been added: (a) dynamic root growth, (b) non-uniform root distribution and water uptake, (c) effect of water stress on plant water uptake, and (d) soil evaporation. Following Warrick’s original model, the extended model ( ALS.f) was also programmed using Fortran 77. See https://zenodo.org/record/42663. To facilitate further development, variables (array variables and non-array variables) used in the original program AL.f, as well the extended program ALS.f, are listed in Supplementary material 1 (in four tables). The subroutine SINK() in ALS.f encapsulates various options for root uptake mechanisms. Similar to some other external subroutines, such as THOMAS(), variables inside the subroutine are local, so that they are not listed in the tables of Supplementary material 1. In the following we document major changes made to the program ALS.f:

### Modeling water flow in variably saturated soil

The model AL.f was modified to include the flux update scheme proposed by 23 and was applied in program a2&3.for of 5. The main idea is summarized in the following (variables referred to are included in ALS.f):

• Assume we use the flux upper boundary condition (B_TYPE= 2). At each new time step, the old hydraulic conductivities and soil water capacities are used to find the needed coefficients for solving the tridiagonal system of equations. However, the surface flux is temporarily not considered (C2=0). Then, these coefficients are provided to the Thomas subroutine to find the updated pressure head ( HSTAR). The trial soil water content at the new time ( THETA) is found using the newly computed conductivities based on values of HSTAR as well as the surface flux.
• The trial value of THETA is accepted only if the soil water content at a depth interval, or that for intervals immediately adjacent to this current depth interval, is unsaturated. In such a case, the soil water retention curve is used to update the new pressure head H based on the trial value of THETA.
• Otherwise, the trial value of THETA is rejected, the value of HSTAR is accepted as the updated value for H, and the updated THETA is computed from the soil water retention curve based on HSTAR.

### Testing different ending soil water potentials for optimal root uptake

Detailed information regarding the root uptake sink term is included in Supplementary material 2. In particular, Equation 1 of Supplementary material 2 illustrates the importance of four water pressure heads for root uptake: *h*_1_ marks the anaerobiosis point; *h*_4_ the pressure head at permanent wilting point, and *h*_2_ and *h*_3_ marks the range of pressure head for optimal root uptake. Here we refer to *h*_3_ as the ending water potential for optimal uptake, because root uptake starts to reduce when the soil water potential becomes lower than this point. As different plants might have different sensitivity to a mild water stress, it is useful to test the model’s sensitivity to *h*_3_.

Following 14 and 24, *h*_3_ may be assumed as $h_{3}^{1}=-11\;m$ for a slow transpiration of $T_{p}^{1}=1\;{\text{mm day}}^{-1}$ and $h_{3}^{2}=-5\;m$ for a fast transpiration of $T_{p}^{2}=5\;{\text{mm day}}^{-1}.$ We tested two more possibilities of the *h*_3_ values, namely, $(h_{3}^{1},h_{3}^{2})=(-14,-8)\;m,$ and $(h_{3}^{1},h_{3}^{2})=(-16,-10),$ for $(T_{p}^{1},T_{p}^{2})=(1,5)\;\text{mm }{\text{day}}^{-1}.$ We also assumed that *h*_3_ changed in a linear fashion for intermediate transpiration rates between $T_{p}^{1}=1$ and $T_{p}^{2}=5\;{\text{mm day}}^{-1}),$ using different $h_{3}^{1}$ and $h_{3}^{2}$ values:

$$h_{3}=aT_{p}+h_{3}^{1}-aT_{p}^{1},\;(5)$$

where $a=(h_{3}^{1}-h_{3}^{2})/(T_{p}^{1}-T_{p}^{2}).$ This change is reflected in the new subroutine HTHREE, as shown in ALS.f. This ( Equation 5) is another form of Equation 2 of Supplementary material 2 but with variable $h_{3}^{1}$ and $h_{3}^{2}$.

### Testing different functions for root water uptake

The extended program ALS.f also allows testing different root uptake functions with or without compensation mechanisms, such as those demonstrated in 14, 15, 25. In particular, in subroutine SINK(), the model by 12 is used when ICPS= 1; a function by 9 is used when ICPS= 3; and the compensation function of 14 (in combination with Wu’s function) is used when ICPS= 2. The normalized root density function ( *L*_*nrd*_) of 9 has four parameters:

The extended program ALS.f also allows testing different root uptake functions with or without compensation mechanisms, such as those demonstrated in 14, 15, 25. In particular, in subroutine SINK(), the model by 12 is used when ICPS= 1; a function by 9 is used when ICPS= 3; and the compensation function of 14 (in combination with Wu’s function) is used when ICPS= 2. The normalized root density function ( *L*_*nrd*_) of 9 has four parameters:

$$L_{nrd}(z_{r})=R_{1}+R_{2}z_{r}+R_{3}z_{r}^{2}+R_{4}z_{r}^{3},\;(6)$$

where *z*_*r*_ = *z*/ *Z*_*r*_( *t*) is the normalized root density at time *t* with *Z*_*r*_( *t*) the maximum rooting depth (m) at time *t*. The coefficients *R*_1_ through *R*_4_ represent experimentally fitted values for wheat, with *R*_1_ = 2.21, *R*_2_ = -3.72, *R*_3_ = 3.46, and *R*_4_ = -1.87 according to 9. The compensation function proposed by 14 at time *t* has the following form:

$$S_{i}=\frac{\alpha_{i}^{2}L_{nrd,i}^{\lambda }T_{p}}{\Delta z_{i}\sum_{i=1}^{n}\alpha_{i}L_{nrd,i}^{\lambda }},\;(7)$$

where *S*_*i*_ is the actual water uptake from *i*th soil depth increment, *α*_*i*_ is the reduction function for this *i*th depth increment, *L*_*nrd,i*_ the normalized root density function for the *i*th depth increment, Δ *z*_*i*_ the thickness (m) of the *i*th depth increment, and *λ* is a fitting parameter varying from 0.01 to 2.0 according to 14.

### Details of simulation-based searching program

In order to cope with situations in which the information of rooting depth and root distribution pattern is unknown, the program ALS.f was converted into a subroutine and is callable by a searching main program, ALSS.f, which is available at https://zenodo.org/record/42702. This conversion was quite straight forward in Fortran 77 in that we only did three things: (a) we deleted parameters BETA and ZRMAX from the parameter listing; (b) we converted the program ALS.f from a main program to a subroutine using 2 input dummy variables BETA and ZRMAX and 7 output dummy variables; and (c) we let this subroutine be called multiple times using different values of true BETA and true ZRMAX from arrays BETAS() and ZRMAX() within the main program unit and made sure that only the values of selected 7 variables were returned to the main program after each run but all the other information from within the subroutine ALS() is lost.

### Operation

The provided two Fortran programs, ALS.f and ALSS.f, can be run from one of the freely available compilers, such as g95 ( http://www.g95.org/). A short introduction of how to install and use g95 to a PC under Window XP or Windows 7 can be found at: http://www.ems.psu.edu/~young/meteo473/Bootcamp/g95Instructions.pdf

To run ALS.f and ALSS.f, the following input information is needed (see Supplementary material 3):

• Soil physical properties and in particular soil water retention curve and saturated hydraulic conductivity. The exact format of data structure is shown in the supplementary data file ALS.DAT, which follows the template provided by 5. A method for predicting soil water retention curves based on easily measured soil texture and bulk density data is illustrated in 26.
• Initial soil water content profile. This must be provided and put to exact format as shown in supplementary data file INITF.DAT, following the template provided by 5. The measured soil water content must be interpolated so that every depth increment of soil has a water content value in it. One method of data interpolation is outlined in 16 using routines provided in 27 and 28.
• Measured terminating soil water content. This can be the measured soil water content on last day of simulation and must be entered in the format as shown in TERMF.DAT of Supplementary material 3. No interpolation is needed for this file.
• Daily weather data. Daily evapo-transpiration rate, daily average air temperature, daily rainfall, relatively humidity, as well as leaf area index, must be entered in data file WEA2.DAT in the order as shown in the data file. Daily leaf area index can be interpolated based on field measured result, as shown in 16.

There are four output files from ALS.f ( Supplementary material 3):

• OUTS.DAT – Mirrors output from AL.f of 5.
• OUTS2.DAT – Daily soil water potential and water contents for interested soil depths.
• INFS.DAT – Mirrors output from AL.f of 5.
• WUE.DAT – Summary of daily cumulative water loss.

There are seven output files from ALSS.f. They provide the following information based on particular realization of combinations of root distribution shape factor BETAS and the maximum rooting depth ZRMAXS ( Supplementary material 3):

• ARD.DAT – Average relative discrepancy between measured and simulated soil water content throughout the simulation period.
• C_AEVA.DAT – Cumulative soil evaporation throughout the simulation period.
• C_ATRA.DAT – Cumulative transpiration throughout the simulation period.
• WBELOW.DAT – Amount of water to deep drainage out of the specified root zone. This was inherited from AL.f
• WADD.DAT – Amount of water added to the system. This was also inherited from AL.f
• BIGI.DAT – Cumulative infiltration. This was also inherited from AL.f
• PCTDIF.DAT – Percent difference in water balance, defined in 5 as (BIGI-WADD)/WADD*100.

The main difference between the output files of ALS.f and those of ALSS.f is that the outputs from the former are a summary of water balance from the simulation of soil water balance based on one set of root parameters (rooting depth and shape factors), while those from the latter ( ALSS.f) contain the simulation outcomes using many sets of root parameters (see next section for further details).

## Use cases

Programs ALS.f and ALSS.f in their current forms were customized to simulate the soil water dynamics for 111 days from May 14 to September 1, 2009. This is reflected both in the programs and the input data files. To use the model in other locations and other durations, appropriate changes must be made both in a few parameters of the programs and input data files. The two potential uses of the programs are outlined in the following:

• If root distribution parameter BETA and maximum rooting depth ZRMAX are known, then program ALS.f may be used to simulate soil water dynamics with root uptake. Make sure to provide updated values for the major parameter values, such as JMATUR (Julian day number of growth maturity), JTHAW (Julian day number of growth initiation), BETA, and ZRMAX.
• If root distribution parameter BETA and maximum rooting depth ZRMAX are unknown, then it is recommended to first use ALSS.f to find the optimal values for BETA and ZRMAX before using ALS.f to simulate soil water dynamics. Before running ALSS.f, three things must be done:
  1. Check to see if the current value of *h*_3_ (see Equation 1 of Supplementary material 2) is correct by changing, if necessary, the value of parameter IHTH (current default value is set to 1, meaning $(h_{3}^{1},h_{3}^{2})\;=\;(-11,-5)).$ See the preamble of subroutine SINK() for two other options.
  2. Check to see if the parameter of ICPSN is correctly specified. currently, the default value is 1, meaning to use Ojha’s compensation function. Setting the value of ICPSN to 2 will switch to Li’s compensation function, and a value of 3 will invoke Wu’s uptake function (without compensation).
  3. Check the nested DO LOOPs structure to adjust the admissible values for index variable II (currently it is set to have 16 different values for BETA) and JJ (currently it is set to have 22 different values for ZRMAX). At the same time, the following lines in the main program of ALSS.f must be changed to be compatible with the range of index values in the nested DO LOOPS.

BETAS(1)=0.5
      DO I=1,15
        BETAS(I+1)=BETAS(I)+0.25
      END DO
      
      ZRMAXS(1)=0.75
      DO I=1,21
        ZRMAXS(I+1)=ZRMAXS(I)+0.05
      END DO
• Finally, it is recommended to put both the program files and the required input data files in the same folder before running the programs.

## Summary

While plant root water uptake has been implemented in commercial software packages, very few researchers have found the time to document important details of how root uptake can be included in a soil water flow model. This paper tries to fill this knowledge gap by using a simple computer program aided with sample input data sets. Specifically, we modified and extended a soil water flow model of 5 to include plant root water uptake capability. We showed that the extended program has the flexibility of considering different sensitivity of root uptake, as well as different water uptake mechanisms. We also modified the model to search for optimal root parameters based on measured soil water content data. This is relevant for new application in that field data of root development usually is expensive and sometimes difficult to obtain, and the utilization of a searching-based modeling approach will help to identify root parameters for improved water balance characterization, which in turn may help to identify ways to improve crop water use efficiency under field conditions. No menu-driven user interface is provided but the author believes that, under some circumstances of knowledge discovery, it is more efficient to work directly with a well-structured computer program, such as the one described in this paper, than relying on a packaged one.

## Software availability

ALS.f is available from: https://zenodo.org/record/42663

ALSS.f is available from: https://zenodo.org/record/42702

ALS.f archived source code as at the time of publication: http://dx.doi.org/10.5281/zenodo.42663 ^29^

ALSS.f archived source code as at the time of publication: http://dx.doi.org/10.5281/zenodo.42702 ^30^

License: Academic free license ("AFL") v.3.0 http://opensource.org/licenses/afl-3.0.php

## Supplementary material

**Supplementary material 1.** Definition of variables used in the computer programs ALS.f.

This file contains four tables documenting the variables used in AL.f (Supplementary Tables 1 and 3) and those added in ALS.f (Supplementary Tables 2 and 4).

.

**Supplementary material 2.** More details about the root uptake term.

This file documents details of the water uptake term as used in the extended Fortran program ALS.f. It is useful for those who are curious about how the water extraction term is specified in the model.

.

**Supplementary material 3.** Data files for ALS.f and ALSS.f.

This includes (a) four input data files common to ALS.f and ALSS.f, (b) four Output files from ALS.f ( OUTS.DAT, OUTS2.DAT, INFS.DAT, WUE.DAT), (c) seven output files from ALSS.f ( ARD.DAT, C_AEVA.DAT, C_ATRA.DAT, WBELOW.DAT, WADD.DAT, BIGI.DAT, PCTDIF.DAT).
