# Supplementary material for: How to put plant root uptake into a soil water flow model: A documentation with complete computer code
Source: F1000Res. 2022 Aug 8;5:43. Originally published 2016 Jan 8. [Version 2] doi: 10.12688/f1000research.7686.2 (PMC5112575; doi:10.12688/f1000research.7686.2)
Supplement: Peer review history

# Peer review: Reviewer response for version 1

**DOI:** 10.5256/f1000research.8276.r16853

**Published:** 2016-11-11

**Authors:** Hirotaka Saito

**Affiliations:** 1 Graduate School of Agriculture, Tokyo University of Agriculture and Technology, Fuchu, Japan

The article entitled "How to put plant root uptake into a soil water flow model" by Dong is in general well written and technically sound. However when I read this manuscript, I just found it a bit difficult to read as most relevant information such as Feddes function was in a supplemental file. There were no examples in the manuscript either. In my opinion, if the author is providing a tool to evaluate root water uptake, several examples (more than one) need to be included in the manuscript; for example how the choice the model affect soil water dynamics. This fact really makes it difficult to evaluate the manuscript.

- There should be a sink term, S, that is used to account for root water uptake in Eq (1). 
- Figure 1 needs to be upgraded. They get blurred.

Reviewer Expertise:

NA

I confirm that I have read this submission and believe that I have an appropriate level of expertise to confirm that it is of an acceptable scientific standard.

# Peer review: Reviewer response for version 1

**DOI:** 10.5256/f1000research.8276.r11894

**Published:** 2016-10-24

**Authors:** Jun Fan

**Affiliations:** 1 Institute of Soil and Water Conservation, Northwest A&F University, Yangling, China

It is an interesting topic and the text is well written but it is requested some program experience. It is a technical report not fully a scientific paper. It gave sufficient detail information about this model and also some relative programs. If a plant root uptake model with plant growth process which can report LAI or other parameters for ET calculation was put to Soil water flow model, this is more useful. If some cases can be tested and compared with other models, it will make user to use it easily. I fully agreed that it can be published as so far and give more opportunities to test it.

Reviewer Expertise:

NA

I confirm that I have read this submission and believe that I have an appropriate level of expertise to confirm that it is of an acceptable scientific standard, however I have significant reservations, as outlined above.

# Peer review: Reviewer response for version 1

**DOI:** 10.5256/f1000research.8276.r11896

**Published:** 2016-02-04

**Authors:** Brijesh K Yadav

**Affiliations:** 1 Department of Hydrology, Indian Institute of Technology Roorkee, Roorkee, India

The text is nicely written and I have few minor comments as described below.

Some references order is not cited correctly. For example the compensation function in equation 7 is proposed by "15" instead of the cited reference is number "14". Similaraly the rest of the citation numbers needs to be corrected. 

The author has shown the moisture flow equation (Eqn 1) without the sink term. Since the focus of this article  is on plant root uptake,  so sink term should be included in all the governing equations along with their discrete form.

The figure 1 can be improved to make it more readable.

Reviewer Expertise:

NA

I confirm that I have read this submission and believe that I have an appropriate level of expertise to confirm that it is of an acceptable scientific standard.
